# Transcriptome Profiling Revealed Potentially Critical Roles for Digestion and Defense-Related Genes in Insects’ Use of Resistant Host Plants: A Case Study with *Sitobion Avenae*

**DOI:** 10.3390/insects11020090

**Published:** 2020-01-30

**Authors:** Da Wang, Xiaoqin Shi, Deguang Liu, Yujing Yang, Zheming Shang

**Affiliations:** 1 State Key Laboratory of Crop Stress Biology for Arid Areas, Northwest A&F University, Yangling 712100, China; wangda@nwsuaf.edu.cn (D.W.); yyj214812579@126.com (Y.Y.); shangzheming2011@sina.com (Z.S.); 2 College of Plant Protection, Northwest A&F University, Yangling 712100, China; 3 Department of Foreign Languages, Northwest A&F University, Yangling 712100, China; sxq-shi@nwsuaf.edu.cn

**Keywords:** defense-related enzymes, host plant resistance, adaptive differentiation, RNA-seq, wheat, barley

## Abstract

Using host plant resistance (HPR) in management of insect pests is often environmentally friendly and suitable for sustainable development of agricultural industries. However, this strategy can be limited by rapid evolution of insect populations that overcome HPR, for which the underlying molecular factors and mechanisms are not well understood. To address this issue, we analyzed transcriptomes of two distinct biotypes of the grain aphid, *Sitobion avenae* (Fabricius), on wheat and barley. This analysis revealed a large number of differentially expressed genes (DEGs) between biotypes 1 and 3 on wheat and barley. The majority of them were common DEGs occurring on both wheat and barley. GO and KEGG enrichment analyses for these common DEGs demonstrated significant expression divergence between both biotypes in genes associated with digestion and defense. Top defense-related common DEGs with the most significant expression changes included three peroxidases, two UGTs (UDP-glycosyltransferase), two cuticle proteins, one glutathione S-transferases (GST), one superoxide dismutase, and one esterase, suggesting their potentially critical roles in the divergence of *S. avenae* biotypes. A relatively high number of specific DEGs on wheat were identified for peroxidases (9) and P450s (8), indicating that phenolic compounds and hydroxamic acids may play key roles in resistance of wheat against *S. avenae*. Enrichment of specific DEGs on barley for P450s and ABC transporters suggested their key roles in this aphid’s detoxification against secondary metabolites (e.g., alkaloids) in barley. Our results can provide insights into the molecular factors and functions that explain biotype adaptation in insects and their use of resistant plants. This study also has significant implications for developing new resistant cultivars, developing strategies that limit rapid development of insect biotypes, and extending resistant crop cultivars’ durability and sustainability in integrated management programs.

## 1. Introduction

In the constantly changing agricultural landscape, effective management of various insect pests often presents huge challenges. Many pest management tactics, such as cultural, biological and chemical tactics, have been developed to meet these challenges. Among different strategies, deployment of host plant resistance can provide many benefits, such as economic savings, promotions of natural and biological control, and a decrease in potentially hazardous insecticide applications [1,2]. Thus, this pest management strategy developed from naturally evolved plant defenses is often environmentally friendly and suitable for sustainable development of agricultural industries, and it can be very valuable in the integrated management of insect pests for various agricultural crops [1,3]. However, some factors, such as efficacy, lack of resistant varieties and rapid evolution of insect populations, can limit the utilization of host plant resistance in insect pest management [2,4]. One of the most serious challenges to the use of resistant crops against various insect pests is the rapid development of new biotypes [5]. For example, a new biotype of the soybean aphid has developed in North America within 5 years after invasion, despite the genetic bottleneck during the invasion process [2]. Multiple biotypes have been constantly found in many phytophagous insects in response to different levels of host plant resistance, such as the rice brown plant hopper (*Nilaparvata lugens*), Hessian fly (*Mayetiola destructor*), and soybean aphid (*Aphis glycines*) [6,7,8].

In the context of host plant resistance, insect biotypes are defined as populations that are able to survive, reproduce on, and cause injury to a cultivated plant resistant to other populations of the same species [2,4]. So, biotypes can be determined by their unique response profiles (i.e., differential survival or fitness) on different plants [2,4]. Particularly, aphids are prone to adapt and overcome host plant resistance and develop different biotypes on variable plants. For example, fifteen sympatric biotypes of the pea aphid have been found on different host species, and they present gradual levels of genetic divergence [9,10]. A study has found at least 17 aphid species with multiple biotypes evolved [11]. This is probably because differential levels of resistance in variable plants present a huge pressure of natural selection that favors host-associated ecological divergence of various aphid populations [12,13]. Increasing evidence has shown that the differential levels of plant resistance to aphids can be attributed to plant secondary chemistry. For example, different levels of hydroxamic acids in cereal crops (e.g., wheat) can play a key role in resistance of crops against a wide range of insects, including aphids [14,15]. Facing harmful effects of plant secondary compounds, aphids have developed a diversity of detoxification systems, such as cytochrome P450 monooxygenases (P450s), glutathione S-transferases (GSTs) and esterases (ESTs) [16,17,18,19]. There are at least two possible means by which detoxification can lead to differential plant resistance and aphid biotype adaptation: (1) detoxification enzymes can be produced in differential ratios and amounts on different resistant plants; (2) gene mutations can occur in the catalytic sites of detoxification enzymes enabling a much more efficient and effective process of detoxification [2]. Activities of the above-mentioned defense-related enzymes in aphids have been extensively studied, but the underlying molecular variation has received little attention [20,21,22,23]. Since host plant-associated differentiation may often involve genes targeted by selection, identification of elevated levels of molecular differentiation between biotypes (or host-associated populations) is an important step towards fully understanding adaptive divergence, as well as the underlying mechanisms of host plant resistance [24]. Research in this respect can not only expand our understanding of insect–plant interactions, but also help to improve the efficacy, durability, and sustainability for the use of host plant resistance in management of insect pests [2]. So far, such research is still scarce, and molecular factors and functions underlying the use of resistant plants by different aphid biotypes (differential adaptation to different resistant plants) are not well understood.

The English grain aphid, *Sitobion avenae* (Fabricius) (Hemiptera: Aphididae), is a good model to address this issue. This aphid can feed and survive on many species of the Poaceae, including cereal crops and pasture grasses [3,25,26,27]. These host plants exhibit a wide range of concentrations for secondary metabolites (e.g., phenolic compounds, hydroxamic acids and alkaloids), which are chemical defenses involved in resistance against aphids [28,29]. Our previous studies did find some genetic variation among *S. avenae* populations from different cultivated hosts and geographic areas, which could serve as a possible genetic reservoir for adaptation to differential host plant resistance [30,31,32,33]. In addition, based on their unique performance profiles on resistant wheat and barley cultivars, multiple *S. avenae* biotypes were identified [4]. Significant genetic differentiation was detected among these biotypes (e.g., biotype 1 vs. biotype 3, *F_ST_* = 0.157) based on microsatellite data [33]. Despite this, molecular factors and functions underlying the divergence of biotypes in *S. avenae* are still little understood. In this study, molecular variation in two distinct *S. avenae* biotypes (i.e., biotypes 1 and 3) feeding on resistant host plants is determined by using high-throughput sequencing techniques. The objectives are to: (1) examine potential molecular factors underlying the adaptive divergence of *S. avenae* biotypes; (2) identify functions and genes involved in the use of resistant plants (i.e., adaptation to specific plants) by different *S. avenae* biotypes.

## 2. Materials and Methods

### 2.1. Aphid Sample Collection and Colony Establishment

In our previous study on the identification of *S. avenae* biotypes [4], we found that biotype 3 was characterized by an ability to overcome the resistance of barley (*Hordeum vulgare* L.) cultivars (e.g., Xiyin No.2), but not wheat (*Triticum aestivum* L.) cultivars (e.g., Aikang 58). On the contrary, biotype 1 was able to overcome the resistance of wheat cultivars (e.g., Aikang 58), but not barley cultivars (e.g., Xiyin No.2). In 2016, clones of biotypes 1 and 3 were sampled on wheat and barley, respectively [4]. The two biotypes of *S. avenae* were reared on the plant of origin (i.e., wheat or barley) under a temperature of 22 ± 1 °C, a relative humidity of 65 ± 5%, and a photoperiod of 16:8 (L:D) h. Prior to the following experiments, all aphid clones were maintained under common laboratory conditions for at least three generations for the purpose of minimizing confounding environmental effects.

### 2.2. Fitness Bioassays of Both S. Avenae Biotypes on Wheat and Barley

In order to determine their fitness on wheat and barley, new-born first instar nymphs of both *S. avenae* biotypes were transferred onto single two-leaf stage seedlings (one nymph per seedling) of Aikang 58 (i.e., wheat) and Xiyin No.2 (i.e., barley) planted in 200 mL plastic pots [6 cm in diameter, containing turfy soil mixed with vermiculite and perlite (4:3:1, *v*/*v*/*v*)]. Each plastic pot was well enclosed with a transparent plastic cylinder (6 cm in diameter, 15 cm in height) which had a terylene mesh top for ventilation. Test aphid individuals on both plants, they were kept in growth chambers with the above-mentioned conditions and monitored for molting and reproductive events daily. New-born aphid individuals were counted and then removed from the plant each day. This experiment was terminated on day 10 after the initiation of reproduction for each test aphid individual. Thirty replicates were conducted for each *S. avenae* biotype on each plant (i.e., wheat or barley). The 10 d fecundities (offspring accumulated in 10 days since the initiation of reproduction) for each biotype on both plants were tabulated and analyzed by using one-way ANOVA in SAS [34]. Post-hoc comparisons between treatments were conducted by using Tukey tests at α = 0.05 following significant ANOVA.

### 2.3. RNA Sampling and Sequencing

New-born individuals of biotypes 1 and 3 were transferred onto Aikang 58 and Xiyin No.2 seedlings (one nymph per seedling) at the two-leaf stage. They were kept under the aforementioned laboratory conditions until reaching the adult stage. After that, they were allowed to feed for additional 24 h, and sampled for RNA sequencing. Ten wingless aphid individuals were collected and put into a 1.5 mL RNase-free tube each time. All RNA-seq aphid samples were frozen immediately in liquid nitrogen and stored in a freezer at –80 °C until use in RNA extraction. There were three biological replications for each *S. avenae* biotype on each plant. The total RNA of each sample was extracted with the MiniBEST Universal RNA Extraction Kit (Takara Bio Inc., Dalian, China), and RNase-free DNase I (Takara Bio Inc., Dalian, China) was used to remove the potential genomic DNA contamination of samples. Following the manufacturers’ instructions, the quality and quantity of RNA samples were estimated with a Bioanalyzer 2100 instrument (Agilent Technologies, CA, US) and NanoPhotometer^®^ spectrophotometer (IMPLEN, CA, US).

The cDNA libraries for RNA samples were generated with the NEBNext^®^ UltraTM RNA Library Prep Kit for Illumina (NEB, Beverly, MA, US). The Agilent Bioanalyzer 2100 system (Agilent Technologies, CA, US) was used to check the quality of these cDNA libraries. The paired-end DNA sequencing of all samples was conducted on an Illumina HiSeq 2500 platform (Illumina Inc., San Diego, CA) of Ovidson Gene Technology Co., Ltd., Beijing, China. Raw datasets of the above-mentioned RNA-seq samples have been submitted to the Sequence Read Archive (SRA) database of NCBI (National Center for Biotechnology Information) (NCBI accession ID: PRJNA575173).

### 2.4. Transcriptome Assembly and Annotation

Through filtering out low quality reads (more than 50% of nucleotides with Qphred ≤ 20), and reads with adapters and ambiguous “N” nucleotides >10% in raw datasets, clean datasets were obtained. At the same time, Q20, Q30 and GC-content of these clean datasets were calculated. We pooled clean reads of all samples and conducted the transcriptome assembly in TRINITY (v2.1.1) with default parameters (for details, see [35]). In order to minimize the redundancy effects, the software CD-HIT (v4.6.7) was used to process transcripts with 95% similarity [36]. The longest transcript of each gene was defined as a ‘unigene’ for functional annotation. Seven databases were used to annotate all unigenes: NR (NCBI non-redundant proteins, NCBI BLAST (basic local alignment search tool) 2.2.28+, e-value ≤ 1.0 × 10^−5^), NT (NCBI non-redundant nucleotides, NCBI BLAST 2.2.28+, e-value ≤ 1.0 × 10^−5^), Pfam (protein families, HMMER 3.0 package, hmmscan, e-value ≤ 0.01), KOG (eukaryotic orthologous groups, NCBI Blast 2.2.28+, e-value ≤ 0.001) and Swiss-Prot (a manually annotated and reviewed protein sequence database, NCBI BLAST 2.2.28+, e-value ≤ 1.0 × 10^−5^). Further orthology analyses for unigenes were performed to determine their molecular functions by using the KEGG (Kyoto Encyclopedia of Gene and Genomes) automatic annotation server (KAAS, KEGG Automatic Annotation Server, e-value ≤ 1.0 × 10^−10^) [1], and the resulting KO (KEGG Orthologs) terms were compared between the two biotypes. Gene ontology (GO) terms for each unigene were further processed in Blast2GO v2.5 with a threshold e-value of ≤ 1.0 × 10^−5^ [37].

### 2.5. Analysis of Gene Expression Patterns

The expression levels of each unigene were determined by RSEM v 1.2.3 (bowtie2 default parameters, mismatch = 0) through mapping each clean read to the assembled transcriptome [38]. Based on the obtained matrix of raw counts for each unigene in each sample, the differential expression of genes (DEGs) between two *S. avenae* biotypes on each plant (i.e., wheat or barley) was analyzed with DESeq2 (v 1.10.1) by using default parameters [39]. We identified 3392 and 2246 DEGs between biotypes 1 and 3 on wheat and barley, respectively. DEGs co-existed in aphids feeding on wheat and barley were considered to be common DEGs (i.e., 1528)—otherwise, they were considered wheat-specific DEGs (i.e., 1864) or barley-specific DEGs (i.e., 718). The significance of enrichment of differential expression genes in KEGG pathways was tested by using the KOBAS software [40]. GO term enrichment analysis for these DEGs were analyzed and visualized by using the online tools of GO analysis (https://www.omicshare.com/tools/Home/Soft/gogsea).

### 2.6. Quantitative Real-Time PCR (qRT-PCR)

In order to verify the results of the above-mentioned RNA-seq analysis, the expression of 12 selected genes was examined by using the qRT-PCR (quantitative reverse transcription-polymerase chain reaction) method. The gene NADH (nicotinamide adenine dinucleotide dehydrogenase, c93747_g4) was chosen as the reference gene because of its consistent expression in our study [M (stability measure value) = 0.177, SV (stability value) = 0.039]. Gene-specific primers were designed by using Beacon Designer version 8.0 (Premier Biosoft, Palo Alto, CA). All the primers used are listed in Table S1. PCR reactions were prepared with the SYBR Premix Ex Taq™ Kit (Takara, Dalian, China) according to the manufacturer’s instructions. All qRT-PCR reactions were performed on the Roche LightCycler^®^ 480 II system (Roche Diagnostics Ltd., Rotkreuz, Switzerland), with three biological replicates and two technical replicates for each gene. The qPCR cycling parameters were as follows: 95 °C for 5 min, followed by 40 cycles of 95 °C for 10 s and 60 °C for 30 s. To confirm the homogeneity of the PCR products, reaction stages for establishment of melt curves (95 °C for 5 s, 65 °C for 1min, and 95 °C for 5 s) were also included. The relative expression level of each gene was calculated by using the 2^−ΔΔCt^ method [41]. The relative expression levels and FPKM values of each gene were compared between the two biotypes by using the student *t* test in the software SPSS Statistics 23 (IBM SPSS Statistics Inc., Chicago, IL, USA).

## 3. Results

### 3.1. Fitness Performance of Two S. Avenae Biotypes on Wheat and Barley

**Figure 1:**
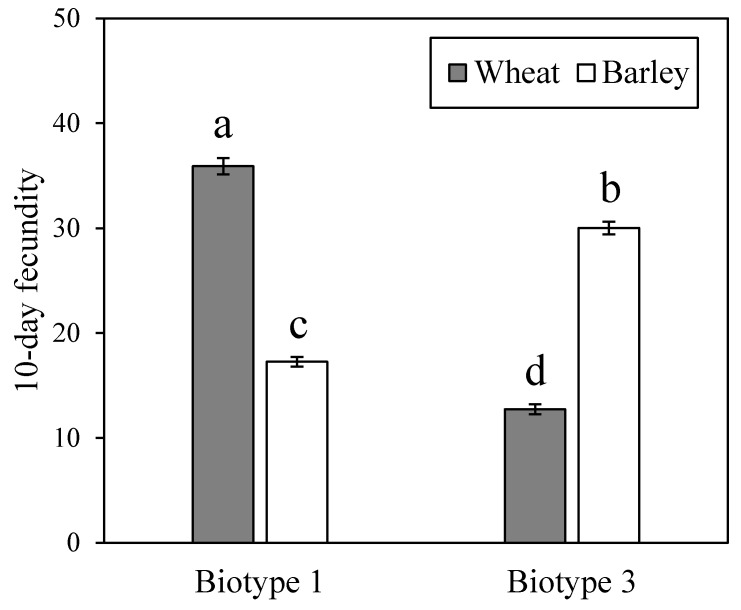
Comparisons of 10 d fecundities for two *Sitobion avenae* biotypes on barley and wheat (different letters on bars indicate significant differences among treatments at α = 0.05, ANOVA followed by Tukey tests).

The 10 d fecundity of *S. avenae* biotype 1 on Aikang 58 (i.e., wheat) (35.9 ± 0.8) was higher than that on Xiyin No.2 (i.e., barley) (17.3 ± 0.5) (Figure 1; *F* = 332.13; df = 3, 116; *p* < 0.001), and biotype 3 showed lower fecundity on wheat (12.7 ± 0.5) than on barley (30.0 ± 0.6). In addition, on wheat, the 10 d fecundity of biotype 1 was higher than that of biotype 3. In contrast, on barley, biotype 3 showed higher fecundity compared with biotype 1. Thus, the differential performance of the two biotypes on wheat and barley clearly suggests their adaptive divergence on both plants.

### 3.2. De Novo Assembly and Transcriptome Annotation

**Figure 2:**
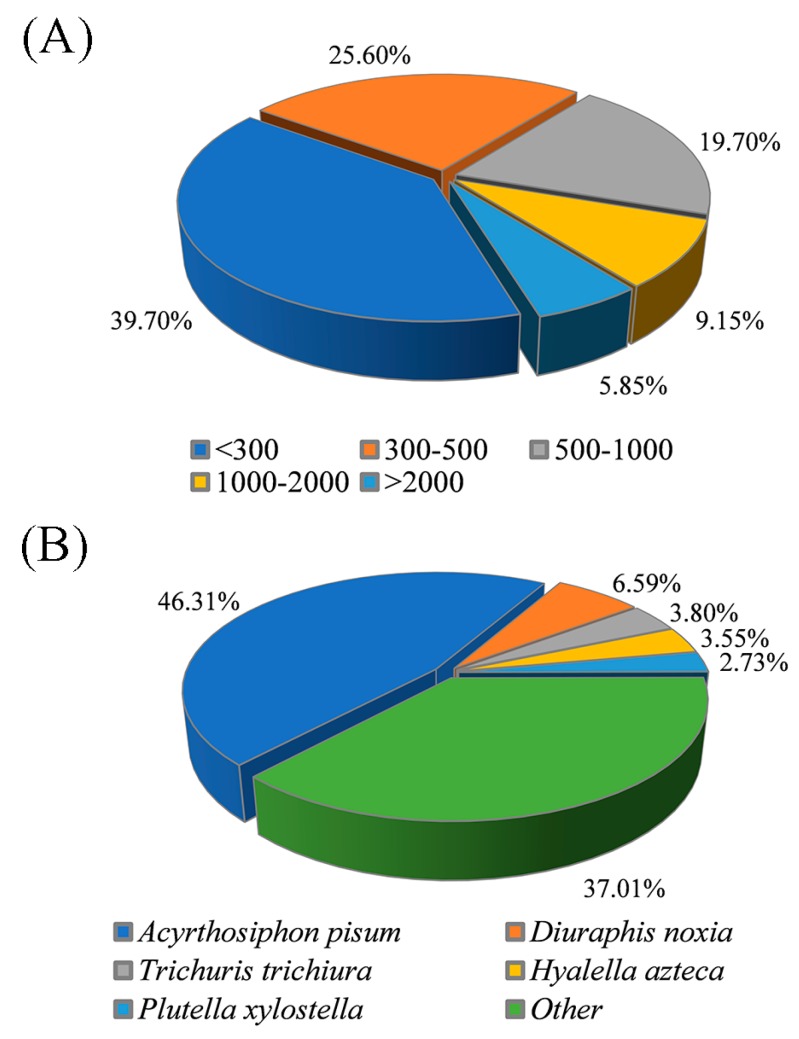
The length distribution of *Sitobion avenae* unigenes in the de novo assembly (**A**), and species distribution of top BLAST hits for the unigenes in the NR database (**B**).

In total, 343,816,452 raw reads and 337,188,919 clean reads were obtained in this study (Table 1). The Q20 of all samples was >98.66%, and the Q30 was >96.18%, showing the high quality of the RNA-Seq analysis. Each sample library was mapped back to the full assembly, with overall alignment rates of 71.67%–73.23%. The de novo assembly yielded 143,058 unigenes. The median length of these unigenes was 358 bp, with a N50 (length N for which 50% of all bases in the assembly are located in a unigene of length < N) of 1012 bp. As shown in Figure 2A, the unigenes of length < 300 bp and >500 bp accounted for 39.70% and 34.70% of the total, respectively, whereas 25.60% of all the unigenes fell between 300 and 500 bp.

For functional annotation, 40.52% (57,961) of all the unigenes matched entries in databases of NR, NT, KO, Swiss-Prot, PFAM, GO or KOG (Table S2). In total, 8420 (5.89%) unigenes had matches in all seven databases. In total, 35,636 (24.91%) and 44,988 (31.45%) unigenes showed hits in the databases NR and NT, respectively. The highest percentage of *S. avenae* unigenes were matched with sequences for *Acyrthosiphon pisum* (46.31%), followed by *Diuraphis noxia* (6.59%), *Trichuris trichiura* (3.80%), *Hyalella azteca* (3.55%), and *Plutella xylostella* (2.73%) (Figure 2B).

### 3.3. Identification of Common and Specific DEGs (Differentially Expressed Genes) and Enrichment Analyses

**Figure 3:**
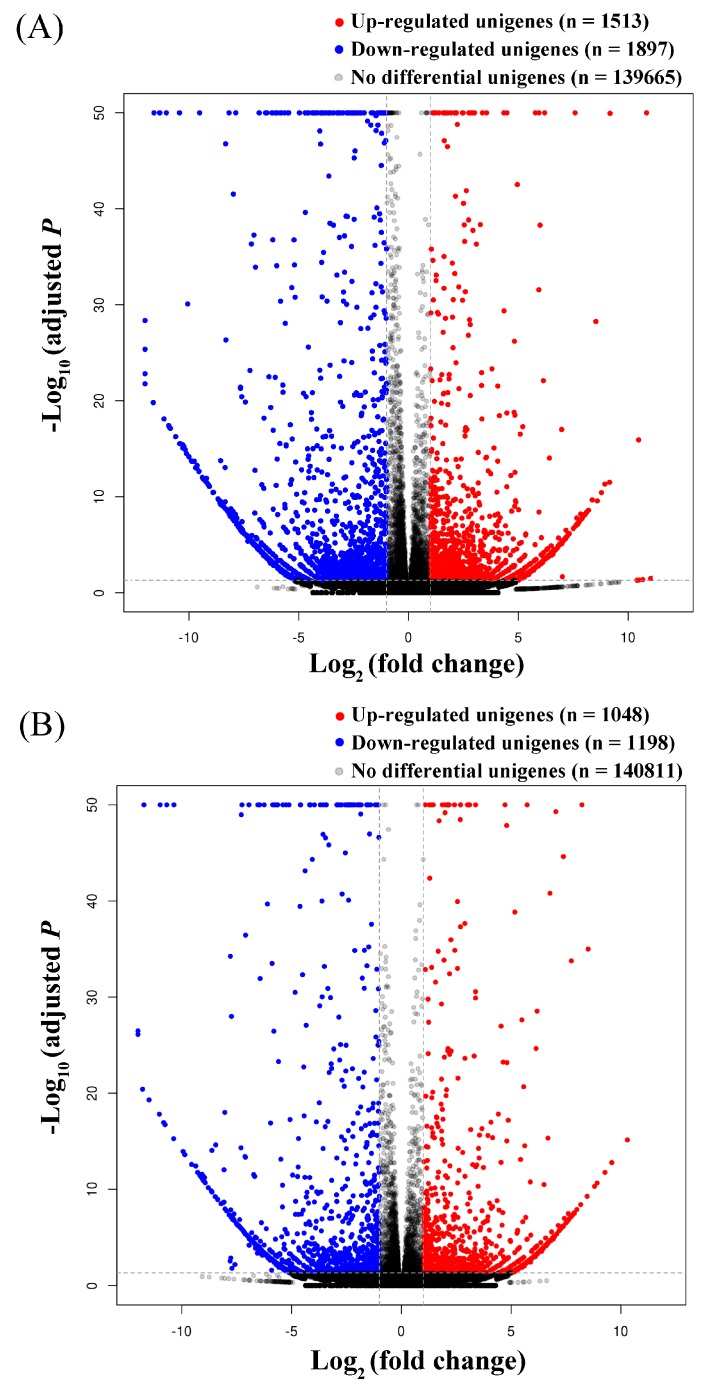
Gene expression changes between two *Sitobion avenae* biotypes (biotype 3 vs. biotype 1) on wheat (**A**) and barley (**B**) (changes between biotypes were considered significantly different if adjusted *p* values < 0.05 and fold change values >2).

**Figure 4:**
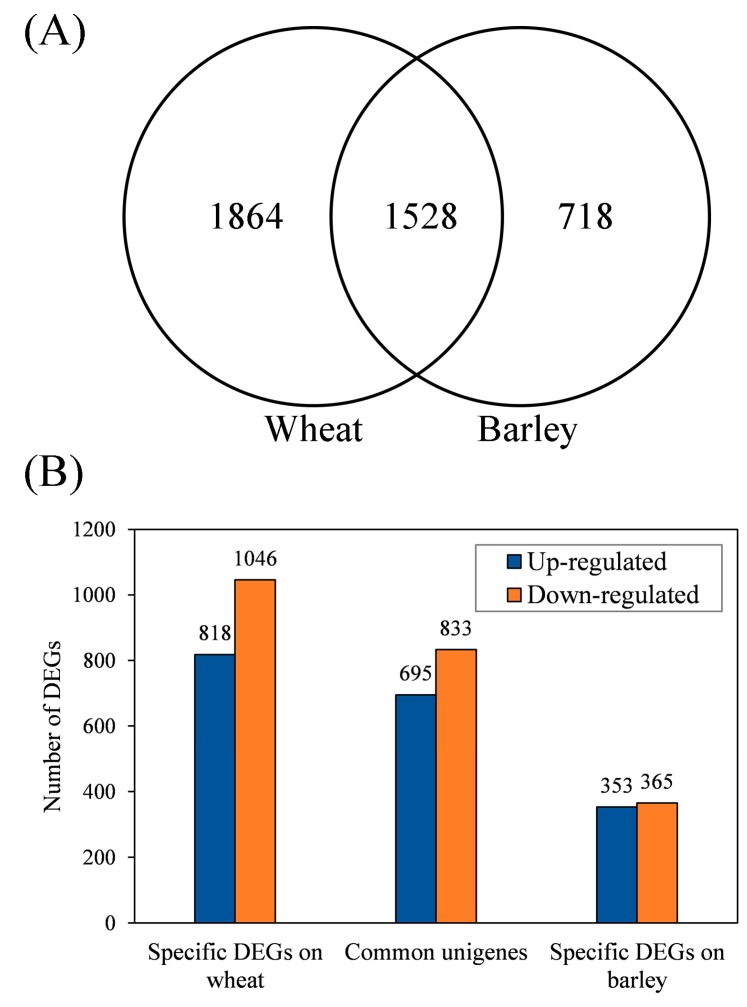
Counts of differentially expressed genes (DEGs; with adjusted *p* values <0.05 and fold change values >2) between *Sitobion avenae* biotypes 1 and 3 (biotype 3 vs. biotype 1) on two plants (**A**) total counts of DEGs; (**B**) the number of up- and down-regulated unigenes).

On wheat, 3392 DEGs (adjusted *p* values <0.05, fold change values >2) were detected between *S. avenae* biotypes 1 and 3 (Figure 3). For these DEGs, 1513 of them were up-regulated, and 1879 were down-regulated in biotype 3 compared to biotype 1 (biotype 3 vs. biotype 1). On barley, 1048 up- and 1198 down-regulated genes (2246 DEGs in total) were detected between the two biotypes (biotype 3 vs. biotype 1). In addition, 1528 DEGs (referred to as common DEGs hereafter) occurred in the two biotypes feeding on both wheat and barley (Figure 4A), and they included 695 up-regulated genes and 833 down-regulated genes (biotype 3 vs. biotype 1, Figure 4B). One thousand eight hundred sixty-four and 718 DEGs (referred to as specific DEGs hereafter) occurred only in the two biotypes feeding on wheat and barley, respectively (Figure 4A). Of the 1864 specific DEGs on wheat, 818 and 1046 genes were up-regulated and down-regulated, respectively (biotype 3 vs. biotype 1) (Figure 4B). Of the 718 specific DEGs on barley, 353 and 365 genes were up-regulated and down-regulated, respectively (biotype 3 vs. biotype 1) (Figure 4B).

**Figure 5:**
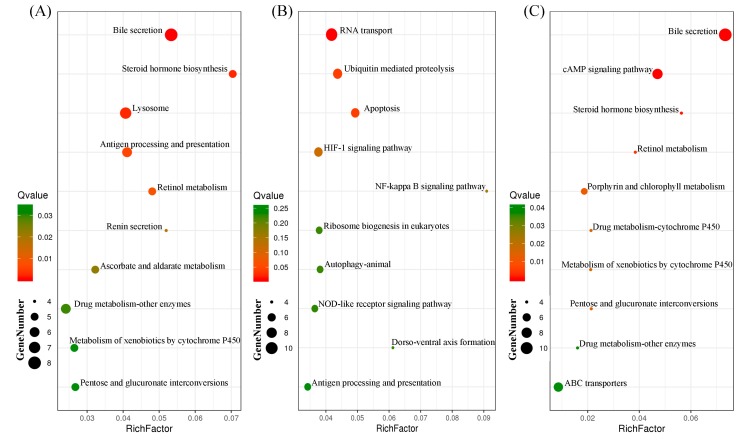
Top 10 enriched KEGG pathway categories for common DEGs (**A**), specific DEGs on wheat (**B**), and specific DEGs on barley (**C**) (bubble color indicates Q-value, bubble size indicates the gene number in KEGG pathways).

Common DEGs were significantly enriched (FDR < 0.05) for GO terms related to activities of proteolysis, peptidase, glucuronosyltransferase, serine-type peptidase, serine hydrolase, glucuronosyltransferase, UDP-glycosyltransferase, and etc. (Table 2). A KEGG pathway enrichment analysis of these common DEGs (Figure 5A) showed that the top 10 enriched pathways were associated with bile secretion (ko04976), steroid hormone biosynthesis (ko00140), lysosome (ko04142), antigen processing and presentation (ko04612), retinol metabolism (ko00830), renin secretion (ko04924), ascorbate and aldarate metabolism (ko00053), drug metabolism-other enzymes (ko00983), pentose and glucuronate interconversions (ko00040), and metabolism of xenobiotics by cytochrome P450 (ko00980). For specific DEGs on wheat, significantly enriched GO terms included regulation of cell proliferation, response to oxidative stress, detoxification, response to toxic substance, response to stress, protein acylation, cysteine-type peptidase activity, peroxidase activity, oxidoreductase activity, and etc (Table 3). In the KEGG analysis of specific DEGs on wheat (Figure 5B), the top 10 enriched pathway categories were RNA transport (ko03013), ubiquitin mediated proteolysis (ko04120), apoptosis (ko04210), HIF-1 signaling pathway (ko04066), NF-kappa b signaling pathway (ko04064), autophagy-animal (ko04140), ribosome biogenesis in eukaryotes (ko03008), NOD-like receptor signaling pathway (ko04621), dorso-ventral axis formation (ko04320), and antigen processing and presentation (ko04612). For specific DEGs on barley, GO terms were significantly enriched (FDR < 0.10) for activities of lipid translocation, phospholipid transport, phospholipid transporter, gamma-glutamyltransferase, lipid transporter, transferase, lysine-tRNA ligase, oxidoreductase, and etc. (Table 4). The top 10 enriched pathway categories of KEGG analysis for specific DEGs on barley (Figure 5C) were bile secretion (ko04976), cAMP signaling pathway (ko04024), steroid hormone biosynthesis (ko00140), retinol metabolism (ko00830), porphyrin and chlorophyll metabolism (ko00860), pentose and glucuronate interconversions (ko00040), drug metabolism-cytochrome P450 (ko00982), metabolism of xenobiotics by cytochrome P450 (ko00980), drug metabolism-other enzymes (ko00983), and ABC transporters (ko02010).

Based on the results of the above-mentioned enrichment analyses, transcriptional changes associated with defense and digestion were further explored. For common DEGs, 16 genes related to digestion were identified, including three serine proteases, five lipases, three phospholipases, two carbohydrases, one maltase, one galactosidase, and one mannosidase (Table 5). Of them, two phospholipases [log2(fold change): 8.35–8.99] and one lipase [log2(fold change): 1.97–2.46] were up-regulated in biotype 1 compared with biotype 3. Forty common DEGs related to defense were also identified, including eight cytochrome P450 monooxygenases (P450s), six UDP-glycosyltransferases (UGTs), three glutathione S-transferases (GSTs), four esterases (ESTs), six peroxidases (PODs), two cuticle proteins (CPs), one heat shock protein (HSP), one superoxide dismutase (SOD), and etc. Among these defense-related DEGs, 13 genes (i.e., four P450s, one GST, two UGTs, the ABC transporter, two alkaline phosphatases, two ESTs and one CP) were up-regulated in biotype 3 compared with biotype 1, whereas the other genes showed higher expression in biotype 1. Among these, the top defense-related common DEGs (biotype 3 vs. biotype 1) with the most significant changes included three PODs [log2(fold change) for c81908_g2: −8.48 to −12.42; for c58802_g1: −5.56 to −5.86; for c86869_g1: −3.57 to −3.63], two CPs [log2(fold change) for c65501_g1: −6.57 to −6.68; for c81826_g1: 4.8 to 5.94], one SOD [log2(fold change): −4.71 to −5.12], one EST [log2(fold change): 3.21 to 4.02], one HSP [log2(fold change): −2.38 to −4.76], two UGTs [log2(fold change) for c91399_g1: −2.18 to −3.32; for c89981_g3: −2.41 to −3.11], and one GST [log2(fold change): 2.84–3.01].

**Figure 6:**
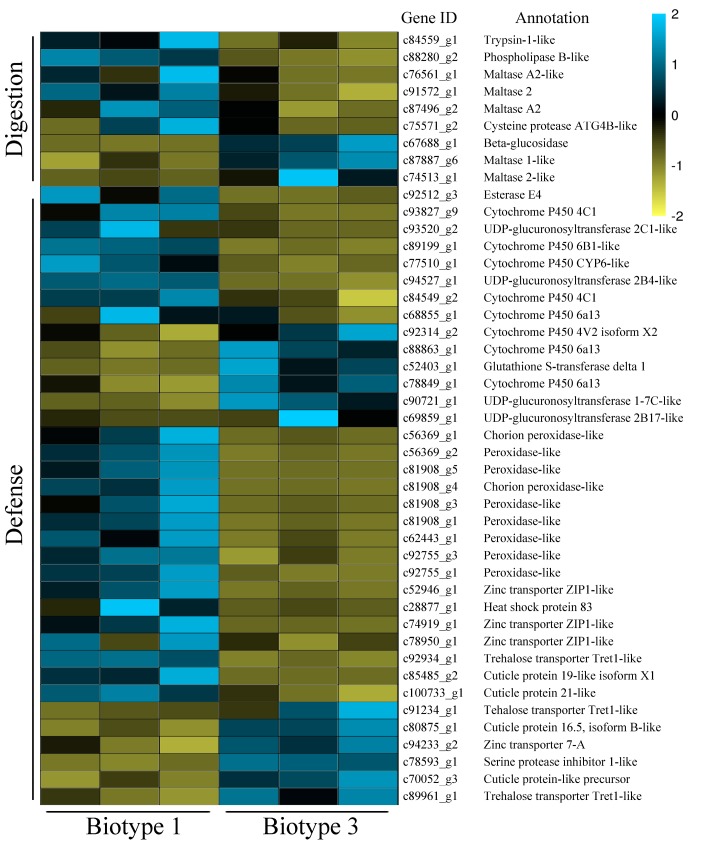
A heatmap of specific DEGs related to digestion and defense in two *Sitobion avenae* biotypes on wheat (genes with expression higher and lower than the mean are indicated by blue and yellow, respectively).

For specific DEGs on wheat, nine genes were annotated to digestion, including five maltases, one *β*-glucosidase, one trypsin, one cysteine protease, and one phospholipase (Figure 6). Except for the two maltases and the phospholipase, all these digestion related DEGs were up-regulated in biotype 1 compared with biotype 3. Thirty-six specific DEGs on wheat were annotated to defense, including eight P450s, four UGTs, one GST, one EST, nine PODs, four CPs, four zinc transporters (ZIPs), one serine protease inhibitor, one HSP, and three trehalose transporters. Among these, five P450s, two UGTs, nine PODs, the EST, three ZIPs, two CPs, one serine protease inhibitor, and one HSP were up-regulated in biotype 1 compared with biotype 3. Among these, the top defense-related specific DEGs (biotype 3 vs. biotype 1) on wheat with the most significant changes included eight PODs [i.e., c81908_g5, c81908_g4, c81908_g1, c81908_g3, c62443_g1, c56369_g1, c56369_g2, c92755_g1; log2 (fold change) values ranged from −6.03 to −11.13] and two UGTs [i.e., c93520_g2 and c69859_g1 with log2 (fold change) values being −5.76 and 6.49, respectively].

**Figure 7:**
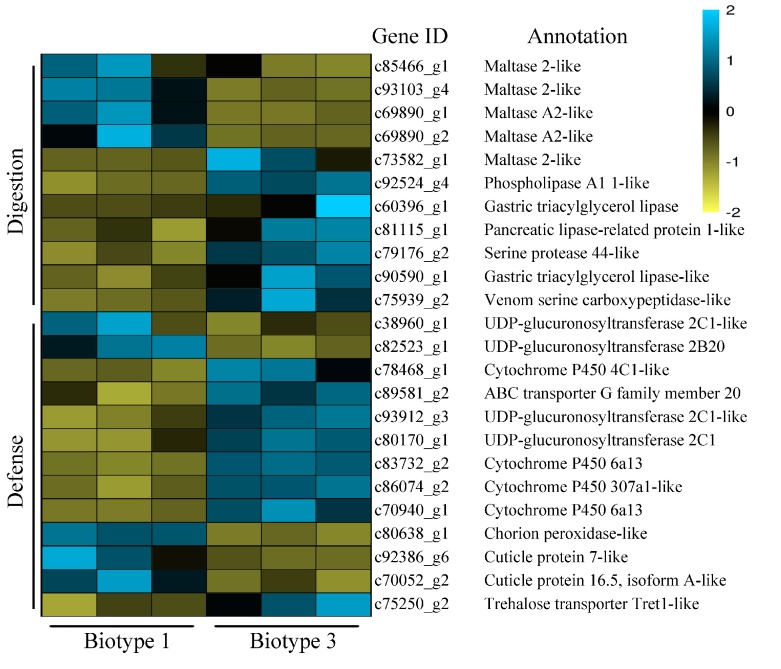
A heatmap of specific DEGs related to digestion and defense in two *Sitobion avenae* biotypes on barley (genes with expression higher and lower than the mean are indicated by blue and yellow, respectively).

For specific DEGs on barley, 11 genes related to digestion were identified, including one serine protease, three lipases, one phospholipase, five maltases, and one serine carboxypeptidase (Figure 7). Among them, all three lipases, one maltase, the serine protease and the serine carboxypeptidase were up-regulated in biotype 3 compared with biotype 1. Thirteen defense-related specific DEGs on barley were also identified, including four P450s, four UGTs, one ABC transporter, one POD, two CPs, and one trehalose transporter. Of them, all four P450s, two UGTs, the ABC transporter, and one trehalose transporter were up-regulated in biotype 3 compared with biotype 1. Among these, the top defense-related specific DEGs (biotype 3 vs. biotype 1) on barley with the most significant changes included two UGTs [i.e., c80170_g1 and c82523_g1 with log2(fold change) values being 5.39 and −1.53, respectively], two P450s [i.e., c70940_g1 and c78468_g1 with log2(fold change) values being 2.71 and 1.58, respectively], and one CP [i.e., c92386_g6, log2(fold change): 2.12].

### 3.4. Validation of RNA-Seq Data by qRT-PCR

**Figure 8:**
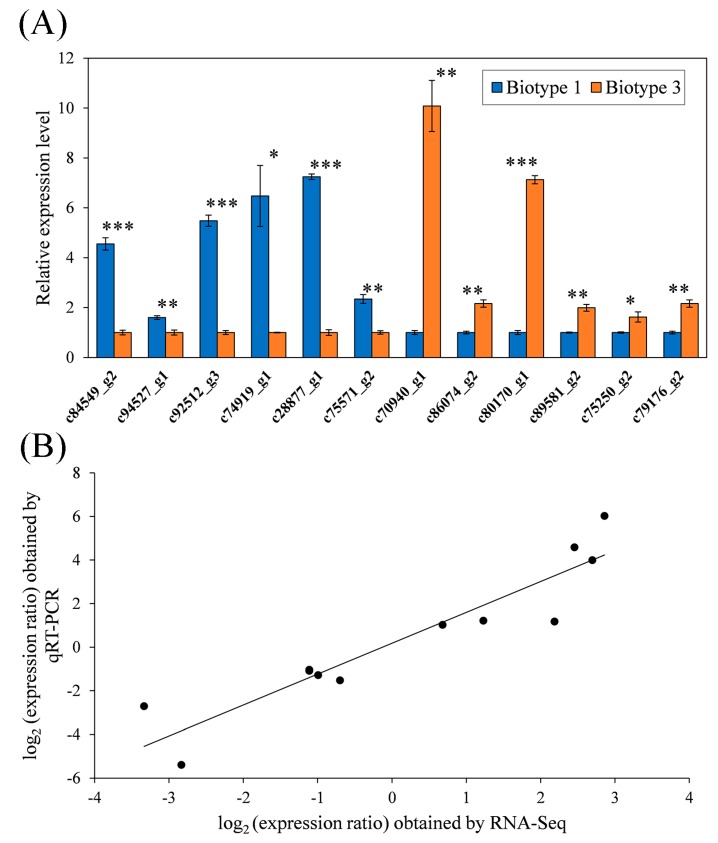
Validation of RNA-Seq results with qRT-PCR of 12 unigenes (**A**), and Pearson’s correlation between fold changes from both qRT-PCR and RNA-Seq (**B**) (c84549_g2, cytochrome P450 4C1; c94527_g1, UDP-glucuronosyltransferase 2B7; c92512_g3, esterase E4; c74919_g1, zinc transporter ZIP1-like; c28877_g1, heat shock protein 83; c75571_g2, cysteine protease ATG4B; c70940_g1, cytochrome P450 6a13; c86074_g2, cytochrome P450 307a1-like; c80170_g1, UDP-glucuronosyltransferase 2C1; c89581_g2, ABC transporter G family member 20; c75250_g2, trehalose transporter Tret1-like; c79176_g2, serine protease 44-like; the first and last six genes were randomly selected from specific DEGs on wheat and barley, respectively; * *p* < 0.05; ** *p* < 0.01; *** *p* < 0.001).

To validate the RNA-seq analysis, the relative expression levels of 12 selected genes were analyzed with RT-qPCR, including six specific DEGs on wheat (i.e., c84549_g2, cytochrome P450 4C1; c94527_g1, UDP-glucuronosyltransferase 2B7; c92512_g3, esterase E4; c74919_g1, zinc transporter ZIP1-like; c28877_g1,→ heat shock protein 83; c75571_g2, cysteine protease ATG4B; c70940_g1) and six specific DEGs on barley (i.e., cytochrome P450 6a13; c86074_g2, cytochrome P450 307a1-like; c80170_g1, UDP-glucuronosyltransferase 2C1; c89581_g2, ABC transporter G family member 20; c75250_g2, trehalose transporter Tret1-like; c79176_g2,→serine protease 44-like). All the selected DEGs showed significant differences in expression between the two *S. avenae* biotypes (Figure 8A). Based on the 12 selected DEGs, the nucleotide differences showed a low genetic divergence between the two biotypes (p-distance = 0.006). In addition, a significant correlation (r = 0.932; *p* < 0.001) was found between datasets of RNA-Seq and qRT-PCR, showing consistency of both analyses (Figure 8B).

## 4. Discussion

### 4.1. Divergence of Aphid Biotypes

Host plant resistance has been used in many integrated management programs for insect pests. However, its use is often limited by the rapid development of various insect biotypes. Molecular researches involving insect biotypes may provide insights into the underlying molecular factors and mechanisms for evolution of biotypes [13,42]. In this study, we used the English grain aphid (*Sitobion avenae*) as a model to address these issues. This aphid was found to have evolved multiple biotypes [4,43]. Among them, biotypes 1 and 3 were showed to have developed a certain degree of specialization on wheat and barley, respectively [4]. The two *S. avenae* biotypes showed differential performance on wheat (Aikang 58) and barley (Xinyin No.2) in this study, confirming their adaptive divergence on wheat and barley reported in our previous study [4]. This is also consistent with the finding that some *S. avenae* clones showed different degrees of specialization on barley and wheat in previous studies [3,30]. We further tested the two *S. avenae* biotypes on both wheat and barley by using transcriptome profiling techniques. A large number of DEGs were identified between the two biotypes when feeding on wheat (i.e., 3392) or barley (i.e., 2246). Interestingly, the majority of them (i.e., 1528) were common DEGs (occurring on both wheat and barley). The enriched GO terms for these common DEGs demonstrated that there had been expression divergence between both biotypes in genes associated with digestion (e.g., peptidase, serine-type peptidase, and serine hydrolase) and defense (e.g., UDP-glycosyltransferase). Similarly, in KEGG enrichment analyses, some digestion-related (e.g., bile secretion) and detoxification-related pathways (e.g., drug and xenobiotics metabolism) were also significantly enriched. Based on these analyses, we identified 16 common DEGs related to digestion, and 40 to defense that could be involved in the biotype divergence process in *S. avenae*.

The digestion-related common DEGs encoded five lipases, three phospholipases, two carbohydrases, one maltase, one galactosidase, one mannosidase, and three serine proteases. It makes sense since many of them (e.g., maltase, galactosidase, mannosidase, lipase and phospholipase) are essential digestive enzymes for glycolysis or esterlysis, and critical to the development and reproduction of insects [44,45,46,47,48,49]. Differential expression of a significant number of digestion-related genes was also identified among different biotypes in the whitefly *Bemisia tabaci* and fruit fly *Drosophila patchea* [50,51]. Thus, the differential expression of these digestive enzymes may have significant implications in the adaptation of insect biotypes on different host plants with significant nutritional variation [52,53,54,55]. In addition to their nutritional implications, some proteases, such as the above-mentioned serine proteases, may also be involved in defensive adaptation against proteinase inhibitors of host plants for insects [56,57]. Indeed, proteinase inhibitors of wheat have been proved to have anti-metabolic effects on *S. avenae* midgut proteases [58].

In addition to serine proteases, 40 other defense-related common DEGs were also identified between the two *S. avenae* biotypes. Among these, the top defense-related common DEGs (biotype 3 vs. biotype 1) with the most significant changes included three peroxidases (PODs), two UDP-glycosyltransferases (UGTs), two cuticle proteins (CPs), one glutathione S-transferase (GST), one superoxide dismutase (SOD), and one esterase (EST) (Table 5). Previous studies have shown that defensive enzymes can be involved in the differentiation of pea aphid biotypes [13,55]. In addition, differences in the expression of genes related to detoxification were also detected between *Subpsaltria yangi* populations specialized on different host plants with variable compositions and concentrations of secondary plant compounds [59,60]. Therefore, all the above-mentioned defense-related common DEGs may play important roles in the divergence of *S. avenae* biotypes. Based on the 12 selected DEGs (Figure 8), nucleotide differences (p-distance = 0.006) showed little genetic divergence between the two biotypes. However, significant genetic differentiation between the two biotypes was detected based on microsatellite data in our previous study [33]. This makes sense since microsatellites are usually located in neutral regions, and the 12 selected DEGs can occur in conservative regions. In addition, in this study, the 12 selected DEGs showed large differences in expression between the two biotypes, indicating the importance of gene expression and regulatory components in the divergence of *S. avenae* biotypes. Further studies on specific functional roles of these defense-related DEGs and their regulatory components in *S. avenae* will increase our understanding of the phenomenon that biotype 1 had much higher proportions and much wider geographic distributions than biotypes 3 [4], as well as the process of biotype evolution in this aphid.

### 4.2. Molecular Factors Underlying Aphids’ Use of Resistant Host Plants

Understanding the molecular differences among these apparently specialized aphid populations can have significant implications for deployment of host plant resistance in management of various pest aphids. In this study, *S. avenae* biotype 1 showed higher fecundity on wheat than on barley, and the opposite was true for biotype 3, suggesting that biotypes 1 and 3 could overcome the resistance of wheat and barley, respectively. We compared the transcriptomes of both *S. avenae* biotypes on the two plants and found that 1864 and 718 DEGs between both biotypes occurred only on wheat and barley, respectively (referred to as specific DEGs). The enriched GO terms for specific DEGs demonstrated that there were significant expression differences in genes associated with defense on wheat (e.g., those for activities of peroxidases, detoxification, and responses to toxic substance) and on barley (e.g., activities of oxidoreductases and gamma-glutamyltransferases). Similarly, in KEGG enrichment analyses of specific DEGs on barley, detoxification-related pathways (e.g., drug metabolism-cytochrome P450, metabolism of xenobiotics by cytochrome P450, drug metabolism-other enzymes, and ABC transporters) were also significantly enriched.

Our further screening analyses for defensive genes revealed 36 specific DEGs on wheat. Among them, a relatively high number of DEGs were identified for PODs (9), P450s (8), UGTs (4), and CPs (4). On barley, only 13 defensive DEGs were found to be specific, and they included four P450s, four UGTs, two CPs, one ABC transporter, one POD, and etc. Thus, wheat and barley with different levels of resistance to either test biotype induced specific expression of many defensive genes in *S. avenae*. For example, wheat induced expression changes for nine specific PODs, whereas only one POD was specifically induced by barley. The specific expression of defense-related genes in the two *S. avenae* biotypes may reflect differential compositions and contents of secondary metabolites (e.g., hydroxamic acids, alkaloids, phenolic compounds, and proteinase inhibitors) between wheat and barley reported in past studies [61,62,63,64,65,66,67]. Indeed, POD activities in *S. avenae* were found to be positively correlated with concentrations of diet phenolic compounds (e.g., flavonoids and tanins) [16,23]. In this study, three DEGs of PODs did show the most significant upregulation [log2 (fold change) for c81908_g5: 11.13; for c81908_g4: 9.34; for c81908_g1: 9.32] in *S. avenae* biotype 1 on wheat compared to biotype 3. The activity of P450s in *S. avenae* was associated with the concentration of plant hydroxamic acids [21,68,69]. High number of specific DEGs on wheat for PODs and P450s, as well as their high expression changes, may indicate that phenolic compounds and hydroxamic acids play critical roles in resistance of wheat against *S. avenae*. For barley, high concentrations of indole alkaloids (e.g., gramine) have often been found to be associated with host plant resistance to aphids [70,71,72,73,74]. In this study, GO and KEGG enrichment analyses showed that P450s and ABC transporters could be critical in defense of *S. avenae* on barley. Thus, P450s and ABC transporters can be key specific DEGs in metabolism of alkaloids in *S. avenae* on barley.

In addition to conventional detoxification DEGs, some digestive DEGs may also be important in *S. avenae*’s use of resistant plants. For example, as one of the critical digestive enzymes responsible for cellulose degradation and glucose production, β-glucosidase was also found to be involved in aphids’ metabolism of the plant secondary metabolite DIMBOA, which occurred frequently in wheat, instead of barley [75,76,77,78,79]. This explains the finding that the expression changes of β-glucosidase were specifically induced in *S. avenae* feeding on wheat in our study. Some proteases, like serine proteases and cysteine proteases, have both digestive and defensive functions in insects [56,57]. In this study, some cysteine proteases showed specific expression in *S. avene* on wheat, whereas some serine proteases were found to be specifically induced by barley. This suggests that serine and cysteine protease inhibitors can play important roles in resistance to aphids on barley and wheat, respectively. Further studies are needed to determine and verify the functional roles of all the putative key specific DEGs (digestive and/or defensive) in *S. avenae* against corresponding secondary compounds in wheat and barley.

## 5. Conclusions

In summary, this RNA-seq analysis provided a solid foundation toward improving our understanding of the molecular factors and functions underlying *S. avenae*’s adaptation to different resistant host plants and its use of resistant hosts. Our study revealed a large number of digestive and defensive DEGs between *S. avenae* biotypes on both wheat and barley, suggesting that digestive and defensive enzymes could facilitate adaptations of *S. avenae* populations to changes in host plant chemistry. Different numbers of defensive DEGs were specifically induced by wheat and barley, reflecting differential compositions and contents of secondary metabolites (e.g., hydroxamic acids, alkaloids, phenolic compounds, and proteinase inhibitors) between both plants. A number of key defensive DEGs in *S. avenae* were found to be potentially involved in the metabolism of the above-mentioned plant secondary metabolites, and they can play a critical role in *S. avenae*’s use of resistant host plants. Detailed studies are needed to determine the specific differences in the secondary metabolism of test plants, and exact functional roles of the putative key defensive DEGs in aphids’ successful use of resistant plants. In addition, some salivary and odorant-binding proteins (c62817_g1 and c71932_g1) were also detected in common DEGs between the two *S. avenae* biotypes, suggesting the significance of these proteins in the differentiation of biotypes in aphids [13,24,42,55]. Further research with these putative key genes will help us to understand the molecular factors and mechanisms that explain virulence and biotype adaptation in insects. It is also a critical step in developing strategies that limit rapid development of insect biotypes and extending resistant crop cultivars’ durability and sustainability in integrated management programs.

## Figures and Tables

**Table 1:** Statistics of RNA-Seq for two *Sitobion avenae* biotypes on wheat and barley.

Parameters	Wheat		Barley	
	Biotype 1	Biotype 3	Biotype 1	Biotype 3
Raw reads	25,989,771–28,117,345	26,056,761–28,490,615	26,108,796–29,031,747	28,413,038–40,153,204
Clean reads	25,482,373–27,506,078	25,567,593–27,982,220	25,669,614–28,512,017	27,900,884–39,420,648
Clean base pairs (Gb)	3.85–4.28	4.19–5.91	3.82–4.13	3.84–4.2
Error (%)	0.01	0.01	0.01	0.01
Q20 (%)	98.76–98.80	98.66–98.79	98.72–98.80	98.70–98.78
Q30 (%)	96.49–96.58	96.18–96.58	96.32–96.59	96.40–96.56
GC (%)	40.04–40.99	40.19–40.34	40.38–40.46	39.70–39.96
Total number of unigenes	143,058			
Total length of unigenes (nt)	95,512,520			
Mean length of unigenes (nt)	358			
N50 of unigenes	1012			
Mapped reads (%)	71.67–72.46	72.80–72.84	72.46–73.23	72.22–73.14

Note: Q20 and Q30, percentage of bases with quality over a Phred score of 20 (i.e., an error rate of 1%) and 30 (i.e., an error rate of 0.1%), respectively; GC, proportion of G and C bases; N50, length N for which 50% of all bases in the assembly are located in a unigene of length < N.

**Table 2:** Gene enrichment analysis of GO terms for common DEGs.

GO	Term Type ^a^	Go Description	DE	Total	*p*-Value	FDR
GO:0006259	P	DNA metabolic process	32	1042	<0.001	0.001
GO:0006508	P	Proteolysis	27	987	<0.001	0.039
GO:0015074	P	DNA integration	17	313	<0.001	0
GO:0005488	F	Binding	178	9929	0.001	0.036
GO:0003676	F	Nucleic acid binding	95	4051	<0.001	0.001
GO:0008233	F	Peptidase activity	30	945	<0.001	0.017
GO:0070011	F	Peptidase activity, acting on L-amino acid peptides	28	847	<0.001	0.017
GO:0004175	F	Endopeptidase activity	17	462	0.001	0.036
GO:0008236	F	Serine-type peptidase activity	13	279	<0.001	0.023
GO:0017171	F	Serine hydrolase activity	13	279	<0.001	0.023
GO:0015020	F	Glucuronosyltransferase activity	7	81	<0.001	0.021
GO:0008194	F	UDP-glycosyltransferase activity	7	107	0.001	0.044
GO:0004523	F	RNA-DNA hybrid ribonuclease activity	5	50	0.001	0.036

Note: All terms significantly enriched with FDR < 0.05; ^a^ F, molecular function; P, biological process.

**Table 3:** Gene enrichment analysis of GO terms for specific DEGs on wheat.

GO	Term Type ^a^	Go Description	DE	Total	*p*-Value	FDR
GO:0006950	P	Response to stress	15	553	<0.001	0.043
GO:0006979	P	Response to oxidative stress	8	140	<0.001	0.02
GO:0018205	P	Peptidyl-lysine modification	7	121	<0.001	0.039
GO:0098754	P	Detoxification	7	136	<0.001	0.042
GO:0098869	P	Cellular oxidant detoxification	7	136	<0.001	0.042
GO:1990748	P	Cellular detoxification	7	136	<0.001	0.042
GO:0009636	P	Response to toxic substance	7	137	<0.001	0.042
GO:0016573	P	Histone acetylation	5	62	<0.001	0.042
GO:0006475	P	Internal protein amino acid acetylation	5	71	<0.001	0.043
GO:0018393	P	Internal peptidyl-lysine acetylation	5	71	<0.001	0.043
GO:0018394	P	Peptidyl-lysine acetylation	5	71	<0.001	0.043
GO:0034655	P	Nucleobase-containing compound catabolic process	5	74	0.001	0.048
GO:0006473	P	Protein acetylation	5	75	0.001	0.048
GO:0043543	P	Protein acylation	5	76	0.001	0.048
GO:0008285	P	Negative regulation of cell proliferation	4	13	<0.001	0.005
GO:0042127	P	Regulation of cell proliferation	4	17	<0.001	0.008
GO:0008283	P	Cell proliferation	4	19	<0.001	0.009
GO:0003676	F	Nucleic acid binding	68	4051	<0.001	0.048
GO:0008234	F	Cysteine-type peptidase activity	10	197	<0.001	0.032
GO:0004601	F	Peroxidase activity	7	118	<0.001	0.045
GO:0016684	F	Oxidoreductase activity	7	130	<0.001	0.048
GO:0004197	F	Cysteine-type endopeptidase activity	5	55	<0.001	0.045

Note: All terms significantly enriched with FDR < 0.05; ^a^ F, molecular function; P, biological process.

**Table 4:** Gene enrichment analysis of GO terms for specific DEGs on barley.

GO	Term Type ^a^	Go Description	DE	Total	*p*-Value	FDR
GO:0034204	P	Lipid translocation	2	8	<0.001	0.039
GO:0045332	P	Phospholipid translocation	2	8	<0.001	0.039
GO:0097035	P	Regulation of membrane lipid distribution	2	8	<0.001	0.039
GO:0015748	P	Organophosphate ester transport	2	12	<0.001	0.055
GO:0015914	P	Phospholipid transport	2	12	<0.001	0.055
GO:0016705	F	Oxidoreductase activity	4	171	0.007	0.078
GO:0005548	F	Phospholipid transporter activity	2	8	0.002	0.030
GO:0003840	F	Gamma-glutamyltransferase activity	2	14	0.003	0.065
GO:0005319	F	Lipid transporter activity	2	20	0.003	0.078
GO:0016755	F	Transferase activity	2	21	0.003	0.078
GO:0004824	F	Lysine-trna ligase activity	2	22	0.004	0.078
GO:0004012	F	Phospholipid-translocating atpase activity	2	8	0.001	0.03

Note: All terms significantly enriched with FDR < 0.10; ^a^ F, molecular function; P, biological process.

**Table 5:** Log_2_ (fold change) of common DEGs related to digestion and defense.

Gene ID	Annotation	Wheat ^a^	Barley ^b^	Gene ID	Annotation	Wheat ^a^	Barley ^b^
		Log_2_ (Fold Change)	Log_2_ (Fold Change)			Log_2_ (Fold Change)	Log_2_ (Fold Change)
c68224_g2	Beta-galactosidase isoform X2	1.67	1.72	c93888_g2	Pancreatic lipase-related protein 1-like	1.1	1.1
c4428_g1	Alpha-mannosidase-like 2	4.06	5.49	c70849_g1	Pancreatic lipase-related protein 2-like	−2.46	−1.97
c86944_g1	Serine protease K12H4.7	2.1	2.14	c88297_g5	Phospholipase YOR022C	−8.35	−8.97
c84329_g2	Serine protease 44-like	2.16	2.09	c88297_g1	Phospholipase YOR022C	−8.35	−8.99
c93201_g1	Serine proteinase stubble-like isoform X1	1.02	1.24	c92524_g1	Phospholipase A1 1-like	1.65	1.9
c90736_g1	Hormone-sensitive lipase	3.51	6.39	c47889_g1	Maltase A2-like	4.84	7.38
c78349_g1	Lipase 3-like	3.34	2.58	c75939_g1	Carboxypeptidase CG4572_56.1	5.26	7.38
c91806_g1	Pancreatic lipase-related protein 2-like	1.6	2.02	c91663_g1	Venom serine carboxypeptidase-like	3.36	4.54
c81636_g2	Cytochrome P450 6a13	1.87	2.77	c75860_g1	Alkaline phosphatase 4	1.67	1.69
c84461_g2	Cytochrome P450 4V2-like	2.64	2.45	c80317_g2	Esterase E4-like	−1.69	−1.54
c94592_g2	Cytochrome P450 4C1-like	1.15	2.17	c89160_g1	Esterase FE4-like isoform X2	−2.37	−1.83
c85949_g2	Cytochrome P450 6a13 isoform X2	1.85	1.42	c73140_g3	Esterase E4-like	3.21	4.02
c82801_g1	Cytochrome P450 4C1-like	−1.85	−1.24	c92686_g1	Esterase FE4-like	2.22	3.04
c92410_g3	Cytochrome P450 6k1	−1.65	−1.42	c88468_g2	Laccase-5	−2.24	−1.49
c81321_g1	Cytochrome P450 6k1-like isoform X1	−1.54	−1.47	c86869_g2	Peroxidase-like	−2.35	−1.47
c92410_g2	Cytochrome P450 6k1-like isoform X2	−2.52	−2.21	c86704_g2	Peroxidase-like	−1.63	−1.69
c94328_g3	Venom carboxylesterase-6-like	−1.44	−1.04	c91728_g2	Peroxidase-like	−1.34	−1.73
c87374_g1	Glutathione S-transferase-like	−1.21	−1.06	c86869_g1	Peroxidase-like isoform X2	−3.63	−3.57
c83325_g1	Glutathione S-transferase omega-1-like	−1.81	−2.29	c58802_g1	Peroxidase	−5.56	−5.86
c89912_g1	Glutathione S-transferase-like	2.84	3.01	c81908_g2	Peroxidase-like	−12.42	−8.48
c89445_g1	UDP-glucuronosyltransferase 2B2	2.01	2.15	c87544_g2	Superoxide dismutase	−4.71	−5.12
c91825_g1	UDP-glucuronosyltransferase 2B7-like	1.15	1.83	c92840_g2	Serine protease inhibitor 5	−1.16	−1.36
c91399_g1	UDP-glucuronosyltransferase 2B4-like	−3.32	−2.18	c65501_g1	Cuticle protein 7-like	−6.68	−6.57
c89981_g2	UDP-glucuronosyltransferase 2B20-like	−2.65	−2.32	c81826_g1	Cuticle protein 7-like	5.94	4.8
c89981_g3	UDP-glucuronosyltransferase 2B1-like	−3.11	−2.41	c69395_g1	Heat shock protein 68-like	−4.76	−2.38
c79169_g2	UDP-glucuronosyltransferase 2B17-like	−1.97	−2.71	c91730_g6	Trehalose transporter Tret1-like	−2.51	−3.55
c72511_g1	ABC transporter G family member 23	2.23	2.8	c19378_g1	Trehalose transporter Tret1	−1.32	−1.13
c93663_g1	Alkaline phosphatase 4	1.62	2.1	c87097_g1	Trehalose transporter Tret1	−1.81	−1.44

^a^ Fold change of gene expression between two biotypes (biotype 3 vs. biotype 1) on wheat; ^b^ Fold change of gene expression between two biotypes (biotype 3 vs. biotype 1) on barley.

## Supplementary Materials

The following are available online at https://www.mdpi.com/2075-4450/11/2/90/s1, Table S1: Primer sequences for selected genes in qRT-PCR, Table S2: Summary statistics of gene annotation in seven databases.

## References

[1] McCarville M.T., O’Neal M.E. (2012). Measuring the benefit of biological control for single gene and pyramided host plant resistance for *Aphis glycines* (Hemiptera: Aphididae) management. J. Econ. Entomol..

[2] Bansal R., Michel A. (2015). Molecular Adaptations of Aphid Biotypes in Overcoming Host-Plant Resistance. Short Views on Insect Genomics and Proteomics.

[3] Gao S., Liu D. (2013). Differential performance of *Sitobion avenae* (Hemiptera: Aphididae) clones from wheat and barley with implications for its management through alternative cultural practices. J. Econ. Entomol..

[4] Wang D., Zhai Y., Liu D., Zhang N., Li C., Shi X. (2019). Identification and Genetic Differentiation of *Sitobion avenae* (Hemiptera: Aphididae) Biotypes in China. J. Econ. Entomol..

[5] Michel A.P., Mittapalli O., Mian M.R. (2011). Evolution of Soybean Aphid Biotypes: Understanding and Managing Virulence to Host-Plant Resistance. Soybean-Molecular Aspects of Breeding.

[6] Sōgawa K. (1982). The rice brown planthopper: Feeding physiology and host plant interactions. Annu. Rev. Entomol..

[7] Ratcliffe R.H., Cambron S.E., Flanders K.L., Bosque-Perez N.A., Clement S.L., Ohm H.W. (2000). Biotype composition of Hessian fly (Diptera: Cecidomyiidae) populations from the southeastern, midwestern, and northwestern United States and virulence to resistance genes in wheat. J. Econ. Entomol..

[8] Kim K.-S., Hill C.B., Hartman G.L., Mian M., Diers B.W. (2008). Discovery of soybean aphid biotypes. Crop Sci..

[9] Peccoud J., Ollivier A., Plantegenest M., Simon J.-C. (2009). A continuum of genetic divergence from sympatric host races to species in the pea aphid complex. Proc. Natl. Acad. Sci. USA.

[10] Peccoud J., Mahéo F., de La Huerta M., Laurence C., Simon J.C. (2015). Genetic characterisation of new host-specialised biotypes and novel associations with bacterial symbionts in the pea aphid complex. Insect Conserv. Divers..

[11] Smith C.M., Chuang W.P. (2014). Plant resistance to aphid feeding: Behavioral, physiological, genetic and molecular cues regulate aphid host selection and feeding. Pest Manag. Sci..

[12] Mullen S.P., Shaw K.L. (2014). Insect speciation rules: Unifying concepts in speciation research. Annu. Rev. Entomol.

[13] Nouhaud P., Gautier M., Gouin A., Jaquiéry J., Peccoud J., Legeai F., Mieuzet L., Smadja C.M., Lemaitre C., Vitalis R. (2018). Identifying genomic hotspots of differentiation and candidate genes involved in the adaptive divergence of pea aphid host races. Mol. Ecol..

[14] Osbourn A.E., Qi X., Townsend B., Qin B. (2003). Dissecting plant secondary metabolism-constitutive chemical defences in cereals. New Phytol..

[15] Sicker D., Schulz M. (2002). Benzoxazinones in Plants: Occurrence, Synthetic Access, and Biological Activity. Studies in Natural Products Chemistry.

[16] Zhang M., Fang T., Pu G., Sun X., Zhou X., Cai Q. (2013). Xenobiotic metabolism of plant secondary compounds in the English grain aphid, *Sitobion avenae* (F.)(Hemiptera: Aphididae). Pestic. Biochem. Physiol..

[17] Sun X.-Q., Zhang M.-X., Yu J.-Y., Jin Y., Ling B., Du J.-P., Li G.-H., Qin Q.-M., Cai Q.-N. (2013). Glutathione S-transferase of brown planthoppers (*Nilaparvata lugens*) is essential for their adaptation to gramine-containing host plants. PLoS ONE.

[18] Birnbaum S.S., Rinker D.C., Gerardo N.M., Abbot P. (2017). Transcriptional profile and differential fitness in a specialist milkweed insect across host plants varying in toxicity. Mol. Ecol..

[19] Huang X., Liu D., Zhang R., Shi X. (2018). Transcriptional Responses in Defense-Related Genes of *Sitobion avenae* (Hemiptera: Aphididae) Feeding on Wheat and Barley. J. Econ. Entomol..

[20] Cai Q.-N., Han Y., Cao Y.-Z., Hu Y., Zhao X., Bi J.-L. (2009). Detoxification of gramine by the cereal aphid *Sitobion avenae*. J. Chem. Ecol..

[21] Loayza-Muro R., Figueroa C.C., Niemeyer H.M. (2000). Effect of Two Wheat Cultivars Differing in Hydroxamic Acid Concentration on Detoxification Metabolism in the Aphid *Sitobion avenae*. J. Chem. Ecol..

[22] Ni X., Quisenberry S.S. (2003). Possible roles of esterase, glutathione S-transferase, and superoxide dismutase activities in understanding aphid-cereal interactions. Entomol. Exp. Appl..

[23] Chrzanowski G., Leszczyński B., Czerniewicz P., Sytykiewicz H., Matok H., Krzyżanowski R., Sempruch C. (2012). Effect of phenolic acids from black currant, sour cherry and walnut on grain aphid (*Sitobion avenae* F.) development. Crop. Prot..

[24] Nouhaud P., Peccoud J., Mahéo F., Mieuzet L., Jaquiéry J., Simon J.C. (2014). Genomic regions repeatedly involved in divergence among plant-specialized pea aphid biotypes. J. Evol. Biol.

[25] Blackman R.L., Eastop V.F. (2000). Aphids on the World’s Crops: An Identification and Information Guide.

[26] Fekih I.B., Jensen A.B., Boukhris-Bouhachem S., Pozsgai G., Rezgui S., Rensing C., Eilenberg J. (2019). Virulence of Two Entomophthoralean Fungi, Pandora neoaphidis and Entomophthora planchoniana, to Their Conspecific (*Sitobion avenae*) and Heterospecific (*Rhopalosiphum padi*) Aphid Hosts. Insects.

[27] Yang Y., Kloos S., Mora-Ramírez I., Romeis J., Brunner S., Li Y., Meissle M. (2019). Transgenic Winter Wheat Expressing the Sucrose Transporter HvSUT1 from Barley does not Affect Aphid Performance. Insects.

[28] Corcuera L., Argandona V., Pena G., Perez F., Niemeyer H. Effect of a Benzoxazinone from Wheat on Aphids. Proceedings of the 5th International Symposium on Insect-Plant Relationships.

[29] Niemeyer H.M. (2009). Hydroxamic acids derived from 2-hydroxy-2 H-1, 4-benzoxazin-3 (4 H)-one: Key defense chemicals of cereals. J. Agric. Food Chem..

[30] Gao S.X., Liu D.G., Chen H., Meng X.X. (2014). Fitness traits and underlying genetic variation related to host plant specialization in the aphid *Sitobion avenae*. Insect Sci..

[31] Huang X., Liu D., Wang D., Shi X., Simon J.-C. (2015). Molecular and quantitative genetic differentiation in *Sitobion avenae* populations from both sides of the Qinling Mountains. PLoS ONE.

[32] Wang D., Shi X., Dai P., Liu D., Dai X., Shang Z., Ge Z., Meng X. (2016). Comparison of fitness traits and their plasticity on multiple plants for *Sitobion avenae* infected and cured of a secondary endosymbiont. Sci. Rep..

[33] Wang D., Liu D., Zhai Y., Zhang R., Shi X. (2019). Clonal Diversity and Genetic Differentiation of *Sitobion avenae* (Hemiptera: Aphididae) From Wheat and Barley in China. J. Econ. Entomol..

[34] Institute S. (2017). Base SAS 9.4 Procedures Guide: Statistical Procedures.

[35] Grabherr M.G., Haas B.J., Yassour M., Levin J.Z., Thompson D.A., Amit I., Adiconis X., Fan L., Raychowdhury R., Zeng Q. (2011). Trinity: Reconstructing a full-length transcriptome without a genome from RNA-Seq data. Nat. Biotechnol..

[36] Li W., Godzik A. (2006). Cd-hit: A fast program for clustering and comparing large sets of protein or nucleotide sequences. Bioinformatics.

[37] Conesa A., Götz S., García-Gómez J.M., Terol J., Talón M., Robles M. (2005). Blast2GO: A universal tool for annotation, visualization and analysis in functional genomics research. Bioinformatics.

[38] Li B., Dewey C.N. (2011). RSEM: Accurate transcript quantification from RNA-Seq data with or without a reference genome. BMC Bioinform..

[39] Love J.H., Roper S., Vahter P. (2014). Dynamic complementarities in innovation strategies. Res. Policy.

[40] Xie C., Mao X., Huang J., Ding Y., Wu J., Dong S., Kong L., Gao G., Li C.-Y., Wei L. (2011). KOBAS 2.0: A web server for annotation and identification of enriched pathways and diseases. Nucleic Acids Res..

[41] Livak K.J., Schmittgen T.D. (2001). Analysis of relative gene expression data using real-time quantitative PCR and the 2^-ΔΔCT^ method. Methods.

[42] Eyres I., Jaquiéry J., Sugio A., Duvaux L., Gharbi K., Zhou J.J., Legeai F., Nelson M., Simon J.C., Smadja C.M. (2016). Differential gene expression according to race and host plant in the pea aphid. Mol. Ecol..

[43] Xu Z.-H., Chen J.-L., Cheng D.-F., Sun J.-R., Liu Y., Francis F. (2011). Discovery of English grain aphid (Hemiptera: Aphididae) biotypes in China. J. Econ. Entomol..

[44] Uvarov B.P. (1929). Insect nutrition and metabolism: A summary of the literature. Trans. R. Entomol. Soc. Lond..

[45] Terra W., Ferreira C., De Bianchi A. (1979). Distribution of digestive enzymes among the endo-and ectoperitrophic spaces and midgut cells of Rhynchosciara and its physiological significance. J. Insect Physiol..

[46] Silva C.P., Terra W.R. (1997). α-galactosidase activity in ingested seeds and in the midgut of *Dysdercus peruvianus* (Hemiptera: Pyrrhocoridae). Arch. Insect Biochem. Physiol..

[47] Mehrabadi M., Bandani A.R., Dastranj M. (2014). Salivary digestive enzymes of the wheat bug, *Eurygaster integriceps* (Insecta: Hemiptera: Scutelleridae). Compt. R. Biol..

[48] Terra W.R., Ferreira C. (2012). Biochemistry and molecular biology of digestion. Insect Molecular Biology and Biochemistry.

[49] Weidlich S., Hoffmann K.H., Woodring J. (2015). Secretion of lipases in the digestive tract of the cricket *Gryllus bimaculatus*. Arch. Insect Biochem..

[50] Ye X.-D., Su Y.-L., Zhao Q.-Y., Xia W.-Q., Liu S.-S., Wang X.-W. (2014). Transcriptomic analyses reveal the adaptive features and biological differences of guts from two invasive whitefly species. BMC Genom..

[51] Lang M., Murat S., Clark A.G., Gouppil G., Blais C., Matzkin L.M., Guittard É., Yoshiyama-Yanagawa T., Kataoka H., Niwa R. (2012). Mutations in the neverland gene turned *Drosophila pachea* into an obligate specialist species. Science.

[52] Cooper W.R., Dillwith J.W., Puterka G.J. (2010). Salivary proteins of Russian wheat aphid (Hemiptera: Aphididae). Environ. Entomol..

[53] Nicholson S.J., Hartson S.D., Puterka G.J. (2012). Proteomic analysis of secreted saliva from Russian Wheat Aphid (*Diuraphis noxia* Kurd.) biotypes that differ in virulence to wheat. J. Proteom..

[54] Nicholson S.J., Puterka G.J. (2014). Variation in the salivary proteomes of differentially virulent greenbug (*Schizaphis graminum* Rondani) biotypes. J. Proteom..

[55] Simon J.-C., d’Alencon E., Guy E., Jacquin-Joly E., Jaquiery J., Nouhaud P., Peccoud J., Sugio A., Streiff R. (2015). Genomics of adaptation to host-plants in herbivorous insects. Brief. Funct. Genom..

[56] Broadway R.M. (1997). Dietary regulation of serine proteinases that are resistant to serine proteinase inhibitors. J. Insect Physiol..

[57] Losvik A., Beste L., Mehrabi S., Jonsson L. (2017). The protease inhibitor CI2c gene induced by bird cherry-oat aphid in barley inhibits green peach aphid fecundity in transgenic Arabidopsis. Int. J. Mol. Sci..

[58] Pyati P., Bandani A.R., Fitches E., Gatehouse J.A. (2011). Protein digestion in cereal aphids (*Sitobion avenae*) as a target for plant defence by endogenous proteinase inhibitors. J. Insect Physiol..

[59] Hou Z., Wei C. (2019). De novo comparative transcriptome analysis of a rare cicada, with identification of candidate genes related to adaptation to a novel host plant and drier habitats. BMC Genom..

[60] Liu Y., Qi M., Dietrich C.H., He Z., Wei C. (2019). Comparative sialotranscriptome analysis of the rare Chinese cicada *Subpsaltria yangi*, with identification of candidate genes related to host-plant adaptation. Int. J. Biol. Macromol..

[61] Zúñiga G.E., Argandoña V.H., Niemeyer H.M., Corcuera L.J. (1983). Hydroxamic acid content in wild and cultivated Gramineae. Phytochemistry.

[62] Argandoña V.H., Zuñiga G.E., Corcuera L.J. (1987). Distribution of gramine and hydroxamic acids in barley and wheat leaves. Phytochemistry.

[63] Bollina V., Kushalappa A.C., Choo T.M., Dion Y., Rioux S. (2011). Identification of metabolites related to mechanisms of resistance in barley against *Fusarium graminearum*, based on mass spectrometry. Plant Mol. Biol..

[64] Frank T., Scholz B., Peter S., Engel K.-H. (2011). Metabolite profiling of barley: Influence of the malting process. Food Chem..

[65] Patterson J.H., Newbigin E., Tester M., Bacic A., Roessner U. (2009). Metabolic responses to salt stress of barley (*Hordeum vulgare* L.) cultivars, Sahara and Clipper, which differ in salinity tolerance. J. Exp. Bot..

[66] Yu J., Vasanthan T., Temelli F. (2001). Analysis of phenolic acids in barley by high-performance liquid chromatography. J. Agric. Food Chem..

[67] Irmak S., Jonnala R.S., MacRitchie F. (2008). Effect of genetic variation on phenolic acid and policosanol contents of Pegaso wheat lines. J. Cereal Sci..

[68] Leszczynski B., Dixon A. (1992). Resistance of cereals to aphids: The interaction between hydroxamic acids and glutathione S-transferases in the grain aphid *Sitobion avenae* (F.)(Hom., Aphididae). J. Appl. Entomol..

[69] Leszczynski B., Matok H., Dixon A. (1992). Resistance of cereals to aphids: The interaction between hydroxamic acids and UDP-glucose transferases in the aphid *Sitobion avenae* (Homoptera: Aphididae). J. Chem. Ecol..

[70] Kawada K., Lohar M.K. (1989). Effect of gramine on the fecundity, longevity and probing behaviour of the greenbug, *Schizaphis graminum* (Rondani). Berichte Ohara Instituts Fuer Landwirtsch. Biol. Okayama Univ..

[71] Salas M., Corcuera L. (1991). Effect of environment on gramine content in barley leaves and susceptibility to the aphid *Schizaphis graminum*. Phytochemistry.

[72] Rustamani M.A., Kanehisa K., Tsumuki H., Shiraga T. (1992). Additional observations on aphid densities and gramine contents in barley lines. Appl. Entomol. Zool..

[73] Corcuera L.J. (1993). Biochemical basis for the resistance of barley to aphids. Phytochemistry.

[74] Tuvesson S., Johansson M. (2000). Does indole alkaloid gramine confer resistance in barley to aphid *Rhopalosiphum padi*?. J. Chem. Ecol..

[75] Hofman J., Hofmanova O. (1969). 1, 4-Benzoxazine Derivatives in Plants: Sephadex Fractionation and Identification of a New Glucoside. Eur. J. Biochem..

[76] Niemeyer H.M. (1988). Hydroxamic acids (4-hydroxy-1, 4-benzoxazin-3-ones), defence chemicals in the Gramineae. Phytochemistry.

[77] Cuevas L., Niemeyer H.M., Jonsson L.M. (1992). Partial purification and characterization of a hydroxamic acid glucoside β-D-glucosidase from maize. Phytochemistry.

[78] Givovich A., Sandström J., Niemeyer H., Pettersson J. (1994). Presence of a hydroxamic acid glucoside in wheat phloem sap, and its consequences for performance of *Rhopalosiphum padi* (L.)(Homoptera: Aphididae). J. Chem. Ecol..

[79] Du Fall L.A., Solomon P.S. (2011). Role of cereal secondary metabolites involved in mediating the outcome of plant-pathogen interactions. Metabolites.

