# Supplementary material for: Transcriptome Profiling Revealed Potentially Critical Roles for Digestion and Defense-Related Genes in Insects’ Use of Resistant Host Plants: A Case Study with Sitobion Avenae
Source: Insects. 2020 Jan 30;11(2):90. doi: 10.3390/insects11020090 (PMC7074007; doi:10.3390/insects11020090)
Supplement: Supplementary file 1 [file insects-11-00090-s001.pdf]

Table S1. Primer sequences for selected genes in qRT-PCR

Gene ID	Annotation	Forward primer (5′ → 3′)	Reverse primer (5′ → 3′)
c84549_g2	Cytochrome P450 4C1	TTCCCGATGTCTGTTTCCGT	GCGGTAATGTTCCTAGTTAGGTG
c94527_g1	UDP-glucuronosyltransferase 2B7	GGCTCTGAACCTCACTTGGT	AGTGCTGCAGTGTTTGAAATCAT
c92512_g3	Esterase E4	GGCATTACCTATGGTCCCCG	CAGGCAAAACACTATCGCCG
c74919_g1	Zinc transporter ZIP1-like	TCCAGGAAATCGCACTGACC	AAACGATAGGGCCAGAACGG
c28877_g1	Heat shock protein 83	TGCTGAAGAAGATAATGAGGAAGAG	CAGGATTGCGTGTCCAGATTG
c75571_g2	Cysteine protease ATG4B	CCCACAATCCTTGGGTGTGA	TCGGCAGCATTCCTATTTGC
c70940_g1	Cytochrome P450 6a13	CCAAAACATGGAATTTGGCTAACG	TCAATTTTCTTCGGTTCACGGTT
c86074_g2	Cytochrome P450 307a1-like	TACCACCCGACACATACCCT	TGCCATTTTAGTCTGATGTCGAT
c80170_g1	UDP-glucuronosyltransferase 2C1	TGATCTCTGTCTCGCACACG	GGCAGACGATTAACCACGGA
c89581_g2	ABC transporter G family member 20	ACTTCAGAGTTCCAGAGAAGGG	TAATCCCACGCTGACGCTAC
c75250_g2	Trehalose transporter Tret1-like	TCGAAAAACCCCGATAACTGGTA	CTGAGACATGTATTTCGCGGTC
c79176_g2	Serine protease 44-like	TCCCCATCAAGATCGCAGAA	CCCCAACCACCTGAGTAACA
c93747_g4	NADH	CGAGGAGAACATGCTCTTAGAC	GATAGCTTGGGCTGGACATATAG

Table S2. Summary statistics of gene annotation in seven databases

Database	Number of Unigenes	Percentage (%)
NR	35636	24.91
NT	44988	31.45
KO	22312	15.6
Swiss-Prot	29464	20.6
Pfam	39306	27.48
GO	18536	12.96
KOG	18527	12.95
All Databases	8420	5.89
At least one Database	57961	40.52
Total unigenes	143058	100
